# (2*E*,6*E*)-2,6-Bis(4-ethoxy­benzyl­idene)cyclo­hexa­none

**DOI:** 10.1107/S1600536808034272

**Published:** 2008-10-25

**Authors:** Xiaopeng Shi, Shuqin Li, Zhenzhen Liu

**Affiliations:** a Department of Chemistry and Biology, Xiangfan University, Xiangfan 441053, People’s Republic of China

## Abstract

The title compound, C_24_H_26_O_3_, was prepared by the condensation reaction of 4-ethoxy­benzaldehyde with cyclo­hexa­none. The mol­ecule has crystallographic mirror symmetry and exhibits a butterfly-shaped geometry, with a dihedral angle of 5.46 (1)° between the two benzene rings. Weak inter­molecular C—H⋯π inter­actions help stabilize the crystal structure.

## Related literature

For related structures, see: Du *et al.* (2007 ▶); Liang *et al.* (2007 ▶); Sun *et al.* (2007 ▶); Zhou *et al.* (2007 ▶). For background information, see: Guilford *et al.* (1999 ▶); Ompraba *et al.* (2003 ▶); Yu *et al.* (2000 ▶).
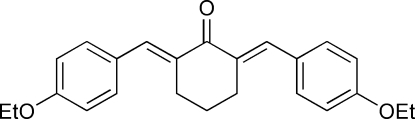


## Experimental

### 

#### Crystal data

C_24_H_26_O_3_
*M* *_r_* = 362.45
Orthorhombic, 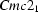

*a* = 24.2516 (6) Å
*b* = 10.8459 (3) Å
*c* = 7.5270 (2) Å
*V* = 1979.83 (9) Å^3^
*Z* = 4
Mo *K*α radiation
μ = 0.08 mm^−1^
*T* = 298 (2) K
0.20 × 0.10 × 0.10 mm

#### Data collection

Bruker SMART 4K CCD area-detector diffractometer
Absorption correction: multi-scan (*SADABS*; Sheldrick, 1997 ▶) *T*_min_ = 0.974, *T*_max_ = 0.992
6002 measured reflections
1026 independent reflections
879 reflections with *I* > 2σ(*I*)
*R*_int_ = 0.121

#### Refinement

*R*[*F* ^2^ > 2σ(*F* ^2^)] = 0.048
*wR*(*F* ^2^) = 0.115
*S* = 1.05
1026 reflections
129 parameters
1 restraint
H-atom parameters constrained
Δρ_max_ = 0.20 e Å^−3^
Δρ_min_ = −0.14 e Å^−3^

### 

Data collection: *SMART* (Bruker, 2001 ▶); cell refinement: *SAINT* (Bruker, 1999 ▶); data reduction: *SAINT*; program(s) used to solve structure: *SHELXS97* (Sheldrick, 2008 ▶); program(s) used to refine structure: *SHELXL97* (Sheldrick, 2008 ▶); molecular graphics: *SHELXTL* (Sheldrick, 2008 ▶); software used to prepare material for publication: *SHELXTL*.

## Supplementary Material

Crystal structure: contains datablocks I, global. DOI: 10.1107/S1600536808034272/bg2205sup1.cif

Structure factors: contains datablocks I. DOI: 10.1107/S1600536808034272/bg2205Isup2.hkl

Additional supplementary materials:  crystallographic information; 3D view; checkCIF report

## Figures and Tables

**Table 1:** Hydrogen-bond geometry (Å, °)

*D*—H⋯*A*	*D*—H	H⋯*A*	*D*⋯*A*	*D*—H⋯*A*
C8—H8⋯*Cg*1^i^	0.93	2.92	3.601 (2)	132

Symmetry code: (i) 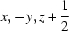. *Cg*1 is the centroid of atoms C6–C11.

**Fig. 1.**
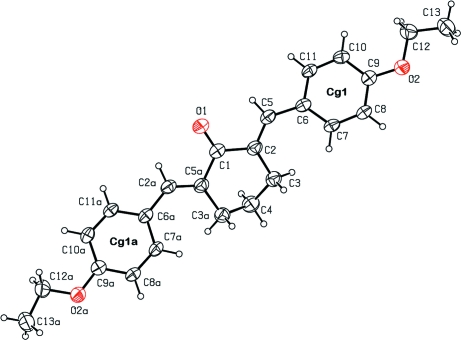
View of the molecule of (I) showing the atom-labelling scheme. Displacement ellipsoids are drawn at the 30% probability level. Symmetry code "a" represents the operation 1-x, y, z

## supplementary crystallographic information

### Comment

The development of highly efficient nonlinear optical crystals is extremely important for laser spectroscopy and laser processing. Bis(arylmethylidene) cycloalkanones are reported to exhibit promising nonlinear optical properties (Yu *et al.*, 2000). In addiiton, these compounds are widely used as building blocks for the synthesis of biologically active heterocycles (Guilford *et al.*, 1999) The title compound C_24_H_26_O_3_ (I) was prepared by the condensation reaction of 4-ethoxybenzaldehyde with cyclohexanone. The molecular structure of (I) is shown in Fig. 1. It has crystallographic mirror symmetry and exhibits a butterfly-shaped geometry. Similar structures have been observed in the related substituted cyclohexanone analogues reported by Ompraba *et al.* (2003) and Sun *et al.* (2007). A dihedral angle of 5.46 (1)° is found between the mean planes of the two benzene rings. Molecules are mainly connected by intermolecular weak C—H···π interactions (Table 1).

### Experimental

The title compound was synthesized as previously described (Sun *et al.*, 2007). 4-Ethoxybenzaldehyde (15.0 g, 0.1 mol) and cyclohexanone (4.9 g, 0.05 mol) were dissolved in 80 ml of ethanol. To this solution, a 10% NaOH aqueous solution (20 ml) was added dropwise with stirring at room temperature. The reaction mixture was stirred for a further 8 h and then poured into a mixture of 100 ml water and diluted hydrochloric acid. The precipitate was filtered and washed thoroughly with water and finally with ethanol. The product was dried at room temperature and crystallized from ethanol to give the title compound as pale yellow solid (12.7 g, yield 70%). Crystals of (I) suitable for X-ray data collection were obtained by slow evaporation of a CH_2_Cl_2_ and MeOH solution in a ratio of 3:2 at 293 K.

### Refinement

All H atoms were positioned geometrically (C—H = 0.93–0.97 Å) and refined using a riding model, with *U*_iso_(H) = 1.2 *U*_eq_(C) (1.5 *U*_eq_(C) for methyl) of the parent atoms. In the absence of significant anomalous dispersion effects, Friedel pairs were averaged.

### Crystal data

**Table:**

C_24_H_26_O_3_	*F*(000) = 776
*M**_r_* = 362.45	*D*_x_ = 1.216 Mg m^−^^3^
Orthorhombic, *C**m**c*2_1_	Mo *K*α radiation, λ = 0.71073 Å
Hall symbol: C2c-2	Cell parameters from 1880 reflections
*a* = 24.2516 (6) Å	θ = 2.7–21.4°
*b* = 10.8459 (3) Å	µ = 0.08 mm^−^^1^
*c* = 7.5270 (2) Å	*T* = 298 K
*V* = 1979.83 (9) Å^3^	Block, colourless
*Z* = 4	0.20 × 0.10 × 0.10 mm

### Data collection

**Table:**

Bruker SMART 4K CCD area-detector diffractometer	1026 independent reflections
Radiation source: fine-focus sealed tube	879 reflections with *I* > 2σ(*I*)
graphite	*R*_int_ = 0.121
φ and ω scans	θ_max_ = 25.5°, θ_min_ = 1.7°
Absorption correction: multi-scan (SADABS; Sheldrick, 1997)	*h* = −24→29
*T*_min_ = 0.974, *T*_max_ = 0.992	*k* = −12→13
6002 measured reflections	*l* = −9→9

### Refinement

**Table:**

Refinement on *F*^2^	Primary atom site location: structure-invariant direct methods
Least-squares matrix: full	Secondary atom site location: difference Fourier map
*R*[*F*^2^ > 2σ(*F*^2^)] = 0.048	Hydrogen site location: inferred from neighbouring sites
*wR*(*F*^2^) = 0.115	H-atom parameters constrained
*S* = 1.05	*w* = 1/[σ^2^(*F*_o_^2^) + (0.0642*P*)^2^] where *P* = (*F*_o_^2^ + 2*F*_c_^2^)/3
1026 reflections	(Δ/σ)_max_ < 0.001
129 parameters	Δρ_max_ = 0.20 e Å^−^^3^
1 restraint	Δρ_min_ = −0.14 e Å^−^^3^

### Special details

**Table:**

Geometry. All e.s.d.'s (except the e.s.d. in the dihedral angle between two l.s. planes) are estimated using the full covariance matrix. The cell e.s.d.'s are taken into account individually in the estimation of e.s.d.'s in distances, angles and torsion angles; correlations between e.s.d.'s in cell parameters are only used when they are defined by crystal symmetry. An approximate (isotropic) treatment of cell e.s.d.'s is used for estimating e.s.d.'s involving l.s. planes.
Refinement. Refinement of *F*^2^ against ALL reflections. The weighted *R*-factor *wR* and goodness of fit *S* are based on *F*^2^, conventional *R*-factors *R* are based on *F*, with *F* set to zero for negative *F*^2^. The threshold expression of *F*^2^ > σ(*F*^2^) is used only for calculating *R*-factors(gt) *etc*. and is not relevant to the choice of reflections for refinement. *R*-factors based on *F*^2^ are statistically about twice as large as those based on *F*, and *R*- factors based on ALL data will be even larger. The authors have merged Friedel pairs before the final refinement. In the absence of anomalous scatterers, no attempt was made to establish the absolute configuration of the title compound.

### Fractional atomic coordinates and isotropic or equivalent isotropic displacement parameters (Å^2^)

**Table:**

	*x*	*y*	*z*	*U*_iso_*/*U*_eq_	
C1	0.5000	0.2069 (4)	0.1013 (7)	0.0752 (13)	
C2	0.44694 (11)	0.1650 (2)	0.1831 (4)	0.0599 (7)	
C3	0.44890 (11)	0.0930 (3)	0.3532 (4)	0.0641 (8)	
H3A	0.4486	0.0056	0.3262	0.077*	
H3B	0.4163	0.1114	0.4231	0.077*	
C4	0.5000	0.1232 (4)	0.4615 (5)	0.0713 (11)	
H4A	0.5000	0.2100	0.4926	0.086*	
H4B	0.5000	0.0755	0.5705	0.086*	
C5	0.40084 (12)	0.1962 (2)	0.0943 (4)	0.0604 (8)	
H5	0.4067	0.2472	−0.0033	0.072*	
C6	0.34301 (12)	0.1637 (2)	0.1252 (3)	0.0534 (7)	
C7	0.32483 (12)	0.0581 (2)	0.2149 (4)	0.0568 (8)	
H7	0.3507	0.0059	0.2663	0.068*	
C8	0.26987 (12)	0.0298 (2)	0.2288 (3)	0.0561 (7)	
H8	0.2590	−0.0412	0.2885	0.067*	
C9	0.23052 (12)	0.1066 (2)	0.1543 (4)	0.0535 (7)	
C10	0.24723 (12)	0.2127 (2)	0.0674 (4)	0.0585 (7)	
H10	0.2212	0.2658	0.0188	0.070*	
C11	0.30222 (11)	0.2389 (2)	0.0536 (4)	0.0572 (7)	
H11	0.3128	0.3101	−0.0062	0.069*	
C12	0.13522 (12)	0.1324 (3)	0.0784 (6)	0.0798 (10)	
H12A	0.1433	0.1310	−0.0477	0.096*	
H12B	0.1331	0.2177	0.1168	0.096*	
C13	0.08165 (14)	0.0681 (4)	0.1142 (7)	0.1005 (14)	
H13A	0.0865	−0.0193	0.1008	0.151*	
H13B	0.0543	0.0965	0.0316	0.151*	
H13C	0.0698	0.0858	0.2332	0.151*	
O1	0.5000	0.2707 (5)	−0.0304 (6)	0.1303 (19)	
O2	0.17725 (8)	0.06933 (16)	0.1745 (3)	0.0673 (6)	

### Atomic displacement parameters (Å^2^)

**Table:**

	*U*^11^	*U*^22^	*U*^33^	*U*^12^	*U*^13^	*U*^23^
C1	0.074 (3)	0.070 (2)	0.082 (3)	0.000	0.000	0.028 (2)
C2	0.0684 (18)	0.0474 (13)	0.0640 (18)	0.0046 (12)	0.0043 (15)	0.0077 (13)
C3	0.0661 (18)	0.0647 (19)	0.0614 (17)	0.0063 (13)	0.0094 (15)	0.0044 (15)
C4	0.085 (3)	0.077 (3)	0.052 (2)	0.000	0.000	0.000 (2)
C5	0.073 (2)	0.0483 (14)	0.0598 (17)	0.0014 (11)	0.0048 (14)	0.0111 (12)
C6	0.0702 (17)	0.0420 (13)	0.0481 (14)	0.0040 (11)	−0.0010 (13)	0.0023 (11)
C7	0.071 (2)	0.0422 (13)	0.0567 (17)	0.0091 (12)	0.0011 (14)	0.0086 (12)
C8	0.0723 (18)	0.0427 (14)	0.0532 (15)	0.0006 (12)	0.0008 (14)	0.0082 (12)
C9	0.0663 (17)	0.0460 (14)	0.0482 (14)	−0.0013 (11)	0.0004 (13)	−0.0063 (12)
C10	0.0701 (19)	0.0449 (14)	0.0605 (16)	0.0083 (12)	−0.0071 (14)	0.0016 (13)
C11	0.0731 (17)	0.0403 (14)	0.0583 (15)	−0.0021 (11)	−0.0034 (15)	0.0083 (11)
C12	0.072 (2)	0.075 (2)	0.092 (3)	0.0082 (16)	−0.008 (2)	0.0095 (19)
C13	0.065 (2)	0.099 (2)	0.138 (4)	0.0051 (17)	−0.004 (2)	0.008 (3)
O1	0.076 (2)	0.170 (4)	0.145 (4)	0.000	0.000	0.110 (4)
O2	0.0661 (13)	0.0624 (10)	0.0733 (14)	−0.0012 (9)	−0.0051 (12)	0.0077 (11)

### Geometric parameters (Å, °)

**Table:**

C1—O1	1.209 (6)	C7—H7	0.9300
C1—C2^i^	1.497 (4)	C8—C9	1.385 (4)
C1—C2	1.497 (4)	C8—H8	0.9300
C2—C5	1.346 (4)	C9—O2	1.362 (3)
C2—C3	1.500 (4)	C9—C10	1.384 (4)
C3—C4	1.519 (4)	C10—C11	1.367 (4)
C3—H3A	0.9700	C10—H10	0.9300
C3—H3B	0.9700	C11—H11	0.9300
C4—C3^i^	1.519 (4)	C12—O2	1.425 (4)
C4—H4A	0.9700	C12—C13	1.499 (5)
C4—H4B	0.9700	C12—H12A	0.9700
C5—C6	1.465 (4)	C12—H12B	0.9700
C5—H5	0.9300	C13—H13A	0.9600
C6—C11	1.391 (4)	C13—H13B	0.9600
C6—C7	1.400 (4)	C13—H13C	0.9600
C7—C8	1.372 (4)		
			
O1—C1—C2^i^	120.73 (18)	C6—C7—H7	119.1
O1—C1—C2	120.73 (18)	C7—C8—C9	120.3 (2)
C2^i^—C1—C2	118.5 (4)	C7—C8—H8	119.9
C5—C2—C1	115.7 (3)	C9—C8—H8	119.9
C5—C2—C3	125.5 (3)	O2—C9—C10	125.3 (2)
C1—C2—C3	118.8 (3)	O2—C9—C8	115.4 (2)
C2—C3—C4	111.8 (3)	C10—C9—C8	119.3 (3)
C2—C3—H3A	109.3	C11—C10—C9	119.6 (3)
C4—C3—H3A	109.3	C11—C10—H10	120.2
C2—C3—H3B	109.3	C9—C10—H10	120.2
C4—C3—H3B	109.3	C10—C11—C6	122.8 (3)
H3A—C3—H3B	107.9	C10—C11—H11	118.6
C3^i^—C4—C3	109.3 (3)	C6—C11—H11	118.6
C3^i^—C4—H4A	109.8	O2—C12—C13	107.8 (3)
C3—C4—H4A	109.8	O2—C12—H12A	110.2
C3^i^—C4—H4B	109.8	C13—C12—H12A	110.2
C3—C4—H4B	109.8	O2—C12—H12B	110.2
H4A—C4—H4B	108.3	C13—C12—H12B	110.2
C2—C5—C6	131.0 (3)	H12A—C12—H12B	108.5
C2—C5—H5	114.5	C12—C13—H13A	109.5
C6—C5—H5	114.5	C12—C13—H13B	109.5
C11—C6—C7	116.3 (3)	H13A—C13—H13B	109.5
C11—C6—C5	118.6 (2)	C12—C13—H13C	109.5
C7—C6—C5	125.1 (3)	H13A—C13—H13C	109.5
C8—C7—C6	121.7 (2)	H13B—C13—H13C	109.5
C8—C7—H7	119.1	C9—O2—C12	118.6 (2)
			
O1—C1—C2—C5	4.1 (7)	C5—C6—C7—C8	−175.8 (3)
C2^i^—C1—C2—C5	−174.1 (3)	C6—C7—C8—C9	−0.4 (4)
O1—C1—C2—C3	−176.0 (5)	C7—C8—C9—O2	179.5 (3)
C2^i^—C1—C2—C3	5.7 (6)	C7—C8—C9—C10	−0.7 (4)
C5—C2—C3—C4	−153.2 (3)	O2—C9—C10—C11	−179.0 (3)
C1—C2—C3—C4	27.0 (4)	C8—C9—C10—C11	1.2 (4)
C2—C3—C4—C3^i^	−59.6 (4)	C9—C10—C11—C6	−0.7 (5)
C1—C2—C5—C6	174.4 (3)	C7—C6—C11—C10	−0.4 (4)
C3—C2—C5—C6	−5.4 (5)	C5—C6—C11—C10	176.5 (3)
C2—C5—C6—C11	158.6 (3)	C10—C9—O2—C12	9.9 (4)
C2—C5—C6—C7	−24.8 (5)	C8—C9—O2—C12	−170.3 (3)
C11—C6—C7—C8	1.0 (4)	C13—C12—O2—C9	176.1 (3)

Symmetry codes: (i) −*x*+1, *y*, *z*.

### Hydrogen-bond geometry (Å, °)

**Table:**

*D*—H···*A*	*D*—H	H···*A*	*D*···*A*	*D*—H···*A*
C8—H8···Cg1^ii^	0.93	2.92	3.601 (2)	132

Symmetry codes: (ii) *x*, −*y*, *z*+1/2.
